# Estrogen receptor beta signaling in CD8^+^ T cells boosts T cell receptor activation and antitumor immunity through a phosphotyrosine switch

**DOI:** 10.1136/jitc-2020-001932

**Published:** 2021-01-18

**Authors:** Bin Yuan, Curtis A Clark, Bogang Wu, Jing Yang, Justin M Drerup, Tianbao Li, Victor X Jin, Yanfen Hu, Tyler J Curiel, Rong Li

**Affiliations:** 1 Department of Biochemistry & Molecular Medicine, School of Medicine & Health Sciences, The George Washington University, Washington, District of Columbia, USA; 2 Department of Medicine, The Mays Cancer Center, University of Texas Health San Antonio, San Antonio, Texas, USA; 3 Department of Molecular Medicine, The Mays Cancer Center, University of Texas Health San Antonio, San Antonio, Texas, USA; 4 Department of Anatomy & Cell Biology, School of Medicine & Health Sciences, The George Washington University, Washington, District of Columbia, USA

**Keywords:** CD8-Positive T-Lymphocytes, Immunity, Immunotherapy

## Abstract

The non-overlapping functions of the two estrogen receptor subtypes, ERα (Estrogen Receptor α)and ERβ (Estrogen Receptor β), in tumor cells have been studied extensively. However, their counterparts in host cells is vastly underinterrogated. Even less is known about how ERα and ERβ activities are regulated in a subtype-specific manner. We previously identified a phosphotyrosine residue (pY36) of human ERβ that is important for tumor ERβ to inhibit growth of breast cancer cells in vitro and in vivo. A role of this ERβ phosphotyrosine switch in regulating host ERβ remains unclear.Conventional gene editing was used to mutate the corresponding tyrosine residue of endogenous mouse ERβ (Y55F) in mouse embryonic stem cells. The derived homozygous mutant *Esr2^Y55F/Y55F^* mouse strain and its wild-type (WT) counterpart were compared in various transplant tumor models for their ability to support tumor growth. In addition, flow cytometry-based immunophenotyping was carried out to assess antitumor immunity of WT and mutant hosts. Adoptive transfer of bone marrow and purified CD8^+^ T cells were performed to identify the host cell type that harbors ERβ-dependent antitumor function. Furthermore, cell signaling assays were conducted to compare T cell receptor (TCR)-initiated signaling cascade in CD8^+^ T cells of WT and mutant mice. Lastly, the ERβ-selective agonist S-equol was evaluated for its efficacy to boost immune checkpoint blockade (ICB)-based anticancer immunotherapy.Disabling the ERβ-specific phosphotyrosine switch in tumor-bearing hosts exacerbates tumor growth. Further, a cell-autonomous ERβ function was defined in CD8^+^ effector T cells. Mechanistically, TCR activation triggers ERβ phosphorylation, which in turn augments the downstream TCR signaling cascade via a non-genomic action of ERβ. S-equol facilitates TCR activation that stimulates the ERβ phosphotyrosine switch and boosts anti-PD-1 (Programmed cell death protein 1) ICB immunotherapy.

Our mouse genetic study clearly demonstrates a role of the ERβ phosphotyrosine switch in regulating ERβ-dependent antitumor immunity in CD8^+^ T cells. Our findings support the development of ERβ agonists including S-equol in combination with ICB immunotherapy for cancer treatment.

## Background

Human ERα and ERβ, which are encoded by different genes (*ESR1* and *ESR2*), mediate the diverse physiological effects of estrogens.^1 2^ Despite sequence homology and similar transcriptional activity, these two ER subtypes exert distinct and even opposite biological functions in cancer. ERα is best known for its role in supporting estrogen-dependent breast tumor growth, whereas ERβ has an antitumor activity in multiple cancer types including breast, prostate, colorectal, ovarian, melanoma and glioma.^1 2^ Research on tumor-related functions of ERα and ERβ has been primarily focused on their tumor-intrinsic activities in regulation of cancer cell behaviors including tumor cell proliferation, migration and invasion. However, emerging evidence suggests that they also play important roles in host cells during cancer development and progression. For example, tumor-extrinsic ERα is implicated in enhancing the immunosuppressive activity of myeloid-derived suppressor cells (MDSC) during ovarian cancer progression.^3^ In support of a tumor-extrinsic antitumor activity of ERβ, syngeneic murine melanoma cells grafted to recipient ERβ knockout (KO) animals grew more robustly than those in wild-type (WT) recipient mice.^4^ More recent studies implicate ERβ in promotion of antitumor immunity.^5 6^ However, how tumor-extrinsic activity of ERβ is regulated in an ER subtype-specific manner remains unclear.

Canonical ERα and ERβ signals activate transcription on their direct binding to the estrogen response element or by tethering other site-specific transcription factors to their cognate target gene loci.^7^ In addition to the nuclear function in transcription activation (genomic action), membrane-associated ERα can elicit rapid signaling events in the cytoplasm within minutes of cellular exposure to extracellular cues such as growth hormones. The latter function of ERα, also called non-genomic action, is distinct from and independent of its canonical transcription activity. It is currently unknown whether a similar non-genomic mechanism is used by ERβ to exert its antitumor activity.

The selective biological effects of ERα and ERβ partly result from their intrinsic differences in protein structure and transcriptional activity. Although the two ER subtypes share a highly homologous central DNA binding domain and carboxyl-terminal ligand-binding domain in the activation function domain 2 (AF2), the more divergent amino-terminal sequence in AF1 domain has been linked to subtype-specific activity. In support, our previous work identified a subtype-specific phosphotyrosine residue in human ERβ AF1 (Y36) that regulates its tumor-intrinsic anti-tumor activity.^8 9^

In the present study, we established a genetically engineered mouse model in which the mouse ERβ equivalent of human Y36 phosphotyrosine residue is mutated to phenylalanine (Y55F). We found that tumors of various types grew significantly faster in ERβ mutant hosts vs. WT control. By adoptive transfer of bone marrow and purified immune cells, we show that ERβ signaling in CD8^+^ immune cells harbors the antitumor function of host ERβ. We further showed that tyrosine phosphorylation of ERβ is stimulated upon T cell receptor (TCR) activation, which in turn enhances the downstream signaling cascade likely via a non-genomic action of ERβ signals. Lastly, we explored the translational utility of rallying ERβ signaling in antitumor immunity by combining the ERβ-selective agonist S-equol with anti-PD-1 immune checkpoint blockade (ICB) immunotherapy.

## Methods

### Animals

All mice had water ad libitum and were fed regular chow. *Rag1^-/-^*, BALB/C, and C57BL/6 CD45.2 and C57BL/6 CD45.1 congenic mice were purchased from the Jackson Laboratory.

Mouse ES clones harboring an ERβ tyrosine-to-phenylalanine (Y55F) mutation were obtained by homologous recombination. Briefly, the targeting vector containing this mutation was introduced into C57BL/6 ES cells via electroporation. Transfected cells were subjected to neomycin selection, and DNA samples from survived clones were analyzed by Southern blotting to identify the correct homologous recombinants. ERβ Y55F mutant mice were generated from the mutant ES cell clone and then crossed with FLP transgenic mouse (Tg(CAG-Flpo)1Afst, MMRRC) to remove the neo marker in vivo. Littermates of WT and homozygous (*Esr2 ^Y55F/Y55F^*) mutant knock-in (KI) mice in the pure C57BL/6 background were generated by intercrossing of heterozygous *Esr2^+/Y55F^* mice. For genome typing, genomic DNA from mouse tail was used as template in PCR reactions with primers comF (5’-CCATCCTACCCTTGGAGCATCG-3’) and wtR (5’-AATCCTACAGTTGGTGTCTCATTGCC-3’) for detection of the *Esr2* WT allele, and primers comF and kiR (5’-CTGACTGATGAAGTTCCTATACTTTC-3’) for detection of the KI allele. The following PCR condition was used: 95°C for 15 min to initially denature DNA, then 35 cycles at 95°C for 20 s, 62°C for 50 s, 72°C for 1 min, and a final extension at 72°C for 7 min. Mice were maintained in a specific pathogen-free facility in accordance with American Association for Laboratory Animal Science guidelines. Littermate animals from different cages were randomly assigned into the experimental groups, which were either cohoused or systematically exposed to other groups’ bedding to ensure equal exposure to all group’s microbiota.

### Cell lines and culture conditions

HEK293T, MC38 colon adenocarcinoma cells, B16F10 melanoma cells, EMT-6 mammary tumor cells and EL4 T lymphoma cells were purchased from ATCC. E0771 mammary tumor cells were purchased from CH3 Biosystems (Cat: 940001). M-Wnt mammary tumor cells were a gift from Dr. Steven Hursting, University of North Carolina. AT-3 mammary tumor cells were generated by Dr. Scott Abrams’s lab at the Roswell Park Comprehensive Cancer Center. ID8agg-Luc ovarian tumor cells were generated by Dr. Tyler Curiel’s lab.^10^ All cell lines were free of *Mycoplasma* and cultured in high glucose DMEM (Thermo Fisher Scientific; Cat: #11965) supplemented with 10% heat-inactivated fetal bovine serum, 1% L-glutamine, 100 µg/mL penicillin and 100 µg/mL streptomycin (P/S, Thermo Fisher Scientific, Cat: #15140122).

### In vivo tumor challenges, treatment and assessment

For tumor studies, mice aged 8–10 weeks were used. M-Wnt (5×10^5^ cells), E0771 (5×10^5^ cells), AT-3 (2×10^5^ cells) and EMT-6 (5×10^5^ cells) tumor cells were inoculated into the fourth mammary pad. B16F10 (0.5×10^6^ cells) and MC38 (5×10^6^ cells) tumor cells were subcutaneously inoculated into the back flank. Tumor volume was measured with calipers on indicated days and calculated as 0.5×length × width.^2^ ID8agg-Luc (4×10^6^) cells were intraperitoneally (i.p.) injected into female mice. In vivo bioluminescence of ID8agg-Luc tumors was assessed with an IVIS Lumina Imaging System (Perkin Elmer; Waltham, MA). Survival was defined as spontaneous death, moribundity, or body weight >130% of baseline (ascites) in ID8agg. Anti-IgG2a (BioXcell, Cat: #BE0146) and anti-PD-1 (BioXcell, Cat: #BE0089) antibodies were administered through i.p. injection at 100 µg/mouse every 3 days starting on day seven after tumor challenge as indicated. S-equol (>99% pure with no detectible R-equol, from Ausio Pharmaceuticals) was fully dissolved in 5% methylcellulose/0.1% Tween 80 on sonication for 5 minutes. It was administered daily by oral gavage at 50 mg/kg beginning on the day of tumor challenge and continued throughout the entire tumor growth experiments.

### Bone marrow chimeras

WT C57BL/6 (CD45.1) recipient males or females (10 mice/transplantation group) were irradiated with 10 Gy total body irradiation as previously described.^11^ Pooled tibial and femoral bone marrow cells from donor WT (CD45.2) or KI (CD45.2) C57BL/6 mice were lysed with RBC lysis buffer. Bone marrow cells from WT or KI mice were retro-orbitally injected into irradiated recipient WT (CD45.1) mice (1×10^7^ cells/recipient mouse). Animals were maintained on trimethoprim-sulfamethoxazole (Hi-Tech Pharmacal) antibiotic water for 1 hour prior and 2 weeks after irradiation, and tumor transplantation of chimeric mice was performed at least 8 weeks after reconstitution. Hematopoietic reconstitution of all animals was verified by flow cytometry of splenocytes at sacrifice.

### Adoptive transfer of CD8^+^ T cells

Total spleen cell suspensions were prepared from 6 to 8 week-old WT and KI male mice. CD8^+^ T cells were isolated from splenocytes by using EasySep Mouse CD8a Positive Selection Kit II (Stemcell Technologies, Catalog #18953). Enriched naïve CD8^+^ T cells were adoptively transferred by intravenous injection (2×10^6^ cells) into *Rag1^-/-^* recipients. B16F10 (0.5×10^6^ cells) melanoma cells were subcutaneously inoculated into the back flank on the following day.

### Flow cytometry

Cells were stained and sorted as previously described,^12^ using LSR II and FACSAria hardware and analyzed by FACSDiva (BD Bioscience) and FlowJo software (FlowJo). Briefly, tumor tissue and tumor-draining lymph node (TDLN) were cut into small pieces and passed through a 70 µm cell strainer to obtain single cell suspension. Dead cells were excluded by staining with Ghost Dye UV 450 (Cat: 13–0868, Tonbo Biosciences). Cells were then blocked with αCD16/32 (Cat: 70–0161, Tonbo Biosciences) at 4°C for 15 min and stained with αCD3 (Cat: 70–0032, Tonbo Biosciences), αCD4 (Cat: 78-0041-82, Invitrogen), αCD8 (Cat: 557654, BD Biosciences), αCD11c (Cat: 117309, BioLegend), αF4/80 (Cat: 123109, BioLegend), αCXCR3 (Cat: 126505, Biolegend) at 4°C for 30 min. For interferon (IFN)γ staining, cells were permeabilized and stained with αIFNγ (Cat: 35–7311, Tonbo).

### CD8 T cell isolation and in vitro activation

CD8^+^ T cells were isolated from the spleens under aseptic conditions. Individual spleens were homogenized to release splenocytes. Cell suspension in 5 mL RPMI 1640 medium was centrifuged (5 min, 300 g at room temperature), supernatant decanted and cell pellet resuspended in the residual volume (approximately 100 µL). Erythrocytes were lysed briefly in 900 µL sterile water (1–2 s) before the addition of 100 µL 10×PBS to restore iso-osmolarity and 5 mL serum-free RPMI 1640 medium. Single-cell suspensions from individual spleens were pooled, filtered through 70 µm and 40 µm cell strainer (Fisher) and counted. CD8^+^ T cells were isolated using magnetic cell sorting by negative selection (MagniSort Mouse CD8 T cell Enrichment Kit, Invitrogen) according to the manufacturer’s instructions. 2×10^6^ isolated CD8^+^ T cells were plated into 24-well plates (Nunc, Thermo Fisher Scientific) pretreated with 10 µg/mL anti-CD3 (clone 145–2 C11, Bio X Cell) and 1 µg/mL anti-CD28 (clone 37.51, Bio X Cell) in PBS overnight at 4°C and washed twice with PBS prior to cell plating.

### Quantitative RT-PCR

Total RNA was isolated from homogenized whole lung tissue using RNeasy (Qiagen). cDNA was synthesized with 1 µg of total RNA using the ImPromII Reverse Transcription System (Promega) and random primers. Quantitative PCR was conducted using the 7900HT Real-Time PCR System (Applied Biosystems), amplified with transcript-specific primers with SYBR Green (Thermo Scientific), according to manufacturer’s instructions. Primer sequences were described previously.^12^

### Immunoblotting

Isolated mouse spleen and bone marrow were lysed in RIPA Lysis and Extraction Buffer. EL4 subcellular fractions were prepared with cell fractionation kit (CST, #9083) following manufacturer’s protocol. Protein amounts were determined by using Pierce BCA Protein Assay Kits (Pierce, Cat: #23225). Primary antibodies were against ERβ mouse mAb (Thermo Fisher, Catalog # MA5-24807), Zap70 (CST, #3165), p-Zap70 (Tyr319)/Syk (Tyr352) (CST, #2717), LAT (CST, #45533), p-LAT (Tyr220) (CST, #20172), Lck (CST, #2752), p-Lck (Tyr505) (CST, #2751), Vimentin (CST, #5741), MEK1/2 (CST, #8727), AIF (CST, #5318), α-Tubulin (CST, #3873). Corresponding secondary antibodies were used. Proteins were detected with ECL SuperSignalTM West Pico PLUS Chemiluminescent Substrate (Thermo Fisher, Cat. #34580).

## ELISA

An equal number of purified CD8^+^ T cells (0.5–2×10^6^) was plated in 24-well plates with or without anti-CD3/CD28 bead addition (Thermo Fisher, Cat. #11 452D). Supernatants were collected after 72 hours, and cytokine production was measured with ELISA kits interleukin (IL)-2 (SM2000, R&D), IFNγ (MIF00, R&D) and tumor necrosis factor (TNF) α (MTA00B, R&D) following manufacturer’s instructions.

### TCGA (The Cancer Genome Atlas) data analysis

For assessing the association of *ESR2* expression with patient survival, patient vital status was used as a surrogate end-point and patients dichotomized by *ESR2* expression. For analyzing *ESR2* expression level versus T cell infiltration in each tumor, the total expression of CD4, CD8α, GZMB (log2 counts per million) was used to assess the infiltration of cytotoxic T-lymphocytes, and correlations were computed vs *ESR2* expression. Correlation of *ESR2* expression levels with survival of breast cancer patients was calculated by level of CD8^+^ T cell infiltration. All patients in the TCGA breast cancer study were divided according to *ESR2* expression (higher or lower than mean expression value of all patients). The correlation of *ESR2* expression level with survival is shown for patients whose tumors had higher (>1 SD) or lower (<1 SD) expression of CD8 [(CD8A+CD8B)/2).^13 14^

### Statistical analysis

Statistical analysis was performed with PrismPad 7 software (GraphPad Prism Software). Data were expressed as mean±SEM. Unpaired Student’s t-test was used for the comparison between two groups. One-way or two-way analysis of variance (ANOVA) followed by the Bonferroni post hoc test was used for the multiple comparisons. Repeated-measure two-way ANOVA (mixed model) followed by the Bonferroni post hoc test was used for the analysis of tumor growth curve. Log-rank test was used to compare Kaplan-Maier curves. A value of p<0.05 was considered significant.

## Results

### Host ERβ signaling inhibits tumor growth

**Figure 1:**
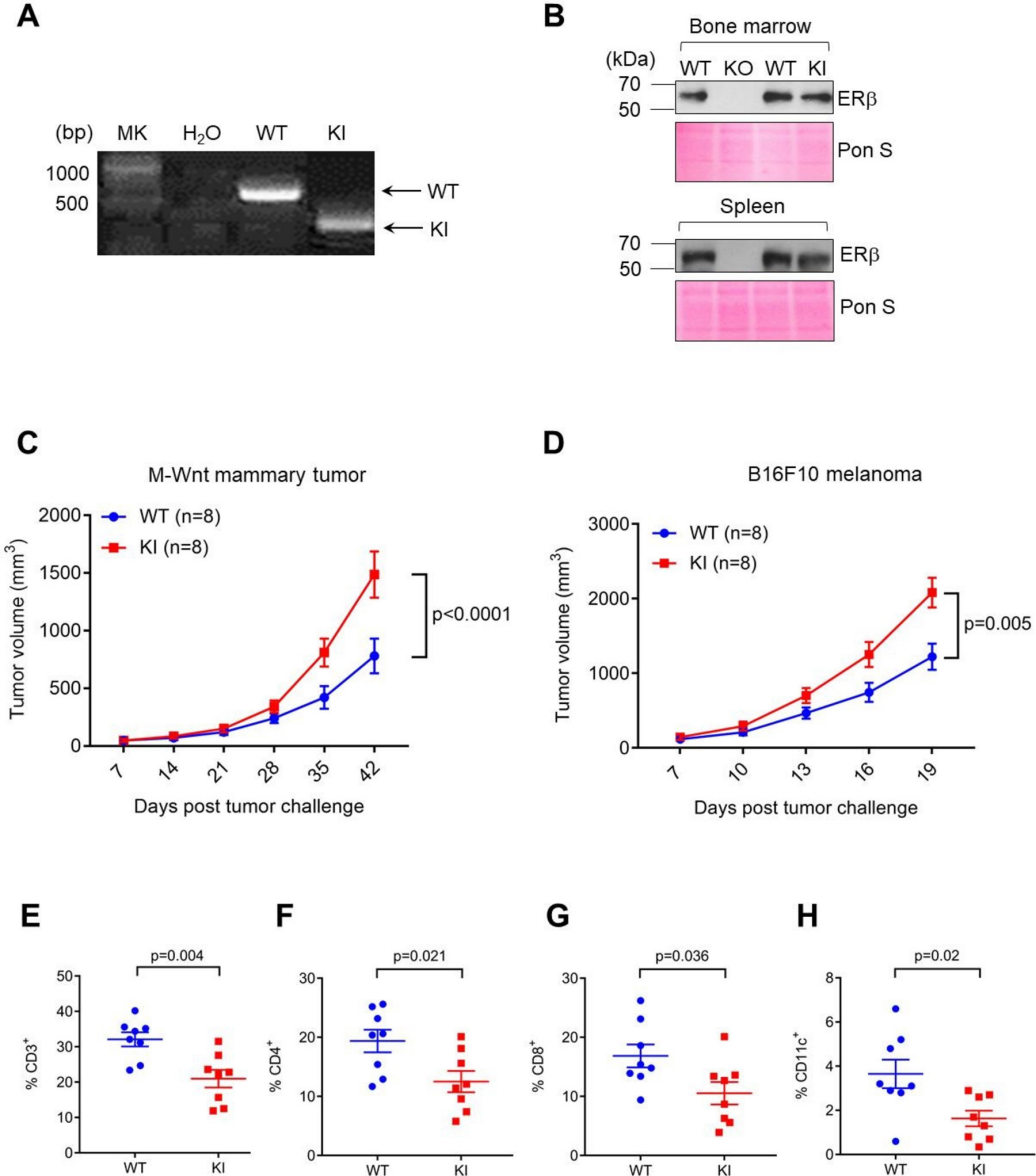
The phosphotyrosine switch is important for host ERβ to inhibit tumor growth. (A) Representative genotyping results for WT and mutant allele of WT and KI mice. (B) ERβ protein levels in mouse bone marrow and spleen (Ponceau S shown as loading control). (C) M-Wnt mammary tumor growth in female WT/KI mice. (D) B16F10 (B16) melanoma growth in WT/KI male mice. (E–H) Flow cytometry of B16 melanoma-infiltrating percentage of CD3^+^ (of CD45^+^), (E), CD4^+^ (of CD45^+^CD3^+^), (F), CD8^+^ (of CD45^+^CD3^+^), (G), CD11c^+^ (of CD45^+^CD3^+^), (H). KI, knock-in; WT, wild-type.

To interrogate tumor-extrinsic function of the ERβ phosphotyrosine switch, we used conventional homologous recombination-based gene editing approach to mutate the tyrosine residue Y55 of endogenous mouse ERβ, which corresponds to Y36 of human ERβ, to phenylalanine (Y55F, (online supplemental figure S1A). The correct genomic alteration at the native *Esr2* locus was confirmed by PCR and sequencing (figure 1A, online supplemental figure S1B). A whole-body homozygous mutant mouse strain (*Esr2^Y55F/Y55F^*) was established in the pure C57BL/6 background and is referred to as knockin (KI) mice herein. Consistent with previously reported ERβ KO mice,^15^ KI mice had no overt developmental defects and were grossly indistinguishable from their WT littermates (online supplemental figure S1C). Survey of ERβ expression in WT, KO and KI mice indicated that the Y55F mutant protein was expressed at levels comparable to WT ERβ in multiple tissues including bone marrow and spleen (figure 1B). Thus, any phenotype associated with KI mice is unlikely due to attenuated ERβ expression.

10.1136/jitc-2020-001932.supp1 Supplementary data

To determine the impact of host ERβ signaling on tumor growth, we transplanted various syngeneic murine tumor cells into WT and homozygous mutant KI mice (*Esr2^Y55F/Y55F^*), including mammary (M-Wnt, figure 1C), colorectal (MC38, online supplemental figure S2A) and melanoma cells (B16F10, figure 1D, online supplemental figure S2B). In all tumor models tested, syngeneic tumors grew more robustly in KI vs. WT counterparts, a trend observed in both male and female cohorts (figure 1D, online supplemental figure S2B). Furthermore, melanoma-bearing KI mice displayed more pronounced lung metastases than WT control (online supplemental figure S2C). However, heterozygous mutant KI mice (*Esr2^WT/Y55F^* or Het) did not confer any appreciable increase in tumor growth vs. WT (online supplemental figure S2D). In concordance with more aggressive tumor growth in KI mice, immunophenotyping of tumor-infiltrating lymphocytes (TILs) indicates that abundance of total CD3^+^ immune cells, CD4^+^ T cells, CD8^+^ T cells and CD11c^+^ dendritic cells was substantially lower in tumors from KI hosts vs WT control (figure 1E–H, online supplemental figure S3A-F). However, F4/80^+^ macrophage abundance was not substantially different between tumor-bearing WT and KI hosts (online supplemental figure S3G). In contrast to the previously reported role of ERα in MDSC,^3^ ERβ signaling did not appear to affect MDSC (CD11b^+^Gr-1^hi^) abundance in tumor-bearing hosts (online supplemental figure S4). Collectively, these data demonstrate that host ERβ phosphotyrosine switch is important for dampening primary and metastatic tumor growth, possibly through rallying antitumor immunity.

10.1136/jitc-2020-001932.supp2 Supplementary data

10.1136/jitc-2020-001932.supp3 Supplementary data

10.1136/jitc-2020-001932.supp4 Supplementary data

#### ERβ signaling in CD8^**+**^ T cells confers host tumor-inhibitory activity

**Figure 2:**
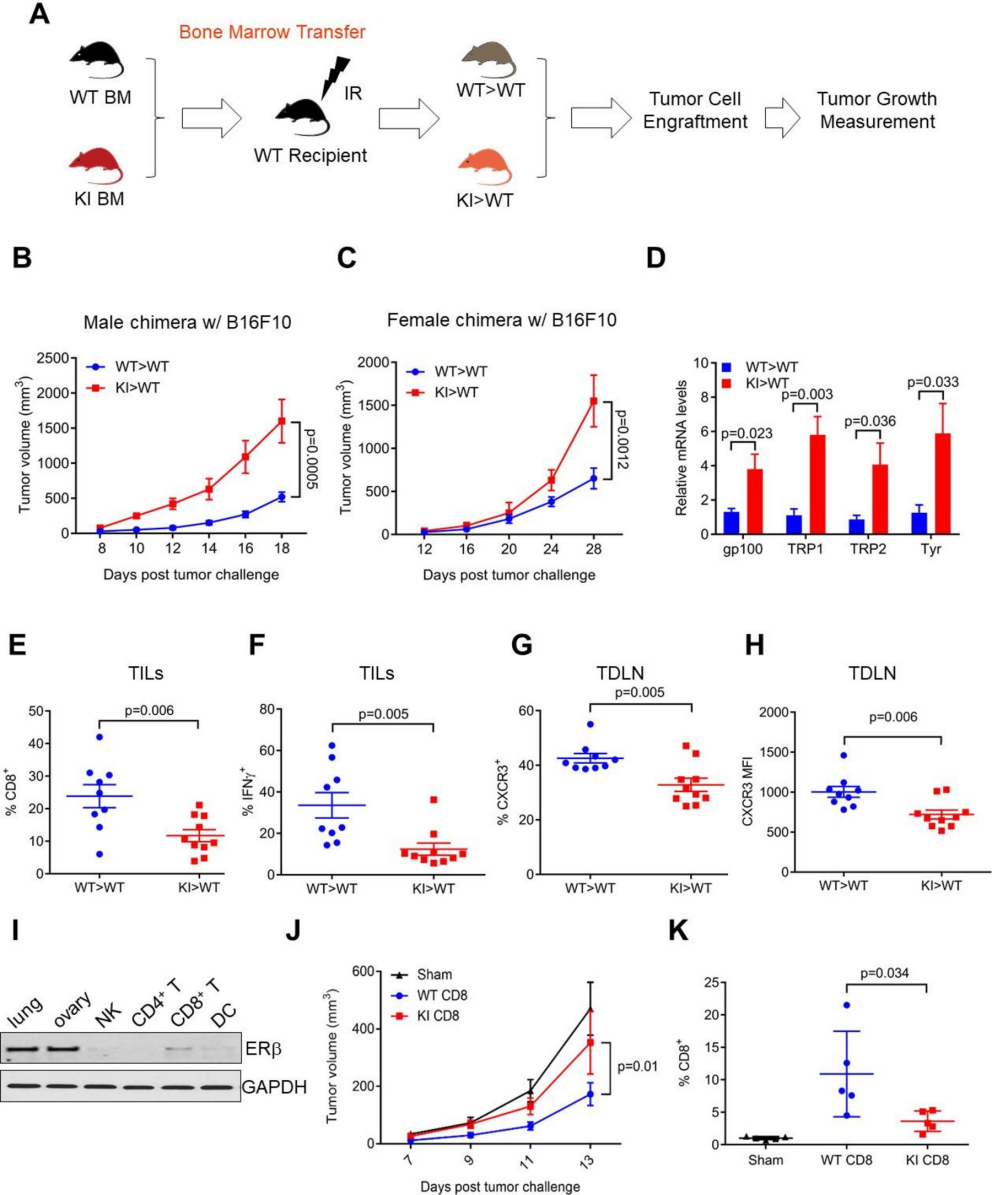
ERβ signaling in CD8^+^ T cells confers tumor inhibition. (A) Schematic representation of the bone marrow adoptive transfer approach used to generate WT >WT or WT >KI mice. A 5-week-old CD45.1^+^ B6.SJL was irradiated (IR) and reconstituted with CD45.2^+^ WT or KI bone marrow. (B, C) B16 melanoma growth in WT >WT and KI >WT male (B) and female (C) chimera mice. (D) qRT-PCR for indicated genes from whole lung lysates of mouse challenged as in (B). (E, F) Flow cytometry of B16 melanoma-infiltrating percentage of CD8^+^ (of CD45^+^CD3^+^), (E), CD8^+^IFNγ^+^ (of CD45^+^CD3^+^), (F) as in (B). (G, H) Flow cytometry was used to analyze the percentage (G) and MFI (H) of CD8^+^CXCR3^+^ cells in TDLN as in (B). (I) ERβ protein levels in purified natural killer (NK), CD4^+^, CD8^+^ T cells, dendritic cells (DC) from spleen, as well as mouse lung and ovary tissue lysate (GAPDH shown as loading controls). GAPDH, Glyceraldehyde 3-phosphate dehydrogenase. (J) B16 melanoma growth in *Rag1^-/-^* mice after adoptive transfer of CD8^+^ T cells from male WT or KI mice. (K) CD8^+^ percentage of B16 melanoma-infiltrating CD45^+^ cell population. IFNγ, interferon-γ; KI, knock-in; MFI, mean fluorescence intensity; TDLN, tumor-draining lymph nodes; TILs, tumor-infiltrating lymphocytes; WT, wild-type.

We next sought to delineate the cellular source of host ERβ signaling in tumor inhibition. Given the significantly reduced TIL abundance in tumors from KI hosts, we generated bone chimeras using recipients of syngeneic WT or KI bone marrow (figure 2A). After confirming successful chimerism in WT>WT and KI>WT mice (online supplemental figure S5A), we injected B16F10 melanoma cells subcutaneously 8 weeks after bone marrow transplant. Tumor growth was significantly faster in male and female KI>WT chimeras (with KI immune cells) vs WT >WT controls (figure 2B–C), suggesting that KI-derived immune cells poorly controlled tumor growth. Consistent with findings from parental KI recipient mice (online supplemental figure S2C), melanoma-associated lung metastasis was greater in male KI>WT mice versus WT>WT control (figure 2D). We peritoneally injected ovarian tumor cells into female chimera hosts and observed significantly shorter median survival of KI>WT mice vs WT>WT controls (30 vs 39 days, p=0.0143, online supplemental figure S5B, C). Thus, our study strongly suggests that ERβ signaling in bone marrow-derived cells influences tumor growth and metastases.

10.1136/jitc-2020-001932.supp5 Supplementary data

In a corollary experiment, we analyzed TILs and TDLN from male B16F10 tumor-bearing chimeric mice. In support of the notion that antitumor immune response of KI mice is compromised, total numbers of tumor-infiltrating CD4^+^ and CD8^+^ T cells were reduced in KI>WT vs WT>WT mice (figure 2E, online supplemental figure S6A). The percentage of IFNγ-producing CD8^+^ cells was significantly lower in tumors from KI>WT versus WT>WT mice (figure 2F), suggesting compromised cytotoxic potency of CD8^+^ T cells in the absence of functional ERβ signaling. Percentage and mean fluorescence intensity of CXCR3 in CD8^+^ T cells were decreased in TDLN of KI>WT mice (figure 2G–H). In addition, activation of dendritic cells, which prime antitumor T cells, was also compromised in KI>WT chimeric mice, as evidenced by their reduced MHC-II expression (online supplemental figure S6B). Furthermore, effector T cells were less activated (CD44/CD62L expression, online supplemental figure S6C) and had reduced additional effector functions in KI>WT chimeras (eg, TNFα and perforin, online supplemental S6D, E)). Paradoxically, T cell PD-1 and Lag3 expression were reduced in tumor-bearing KI mice (online supplemental figure S6F). These markers tend to mark exhausted T cells with reduced antitumor functions, but phenotypic identification of certain activated versus exhausted T cells remains incompletely defined.^16^ Together, these data strongly suggest ERβ-dependent augmentation of antitumor CD8^+^ T cell effector activity and improved tumor immune cell trafficking, which could be from increased dendritic cell activation and/or CD4^+^ T cells as immune mechanisms for ERβ-driven antitumor activity.

10.1136/jitc-2020-001932.supp6 Supplementary data

ERβ mRNA is expressed in various immune cell types including CD8^+^ T cells and dendritic cells, but is barely detectable in CD4^+^ T cells.^17^ To identify the specific immune cell type(s) that confers ERβ-dependent reinforcement of antitumor immunity, we immunoblotted purified primary mouse immune cells and found that among the immune cell types examined, ERβ protein levels were the highest in CD8^+^ cells, followed by those in dendritic cells (figure 2I). To assess a cell-autonomous antitumor effect of ERβ signaling in CD8^+^ T cells, we purified CD8^+^ T cells from WT and KI mice and adoptively transferred them into immunodeficient *Rag1^-/-^* recipient mice. Subsequent tumor study indicates that adoptively transferred WT CD8^+^ T cells conferred significant inhibition of tumor growth as compared with either sham or KI CD8^+^ T cells (figure 2J). When abundance of TILs was examined, we found that number of KI CD8^+^ T cells was substantially lower than WT control (figure 2K). Together, our data provide compelling evidence for a cell-autonomous role of ERβ signaling in CD8^+^ T cells in galvanizing antitumor immunity.

#### Non-genomic action of ERβ in CD8^**+**^ T cells boosts T cell activation

**Figure 3:**
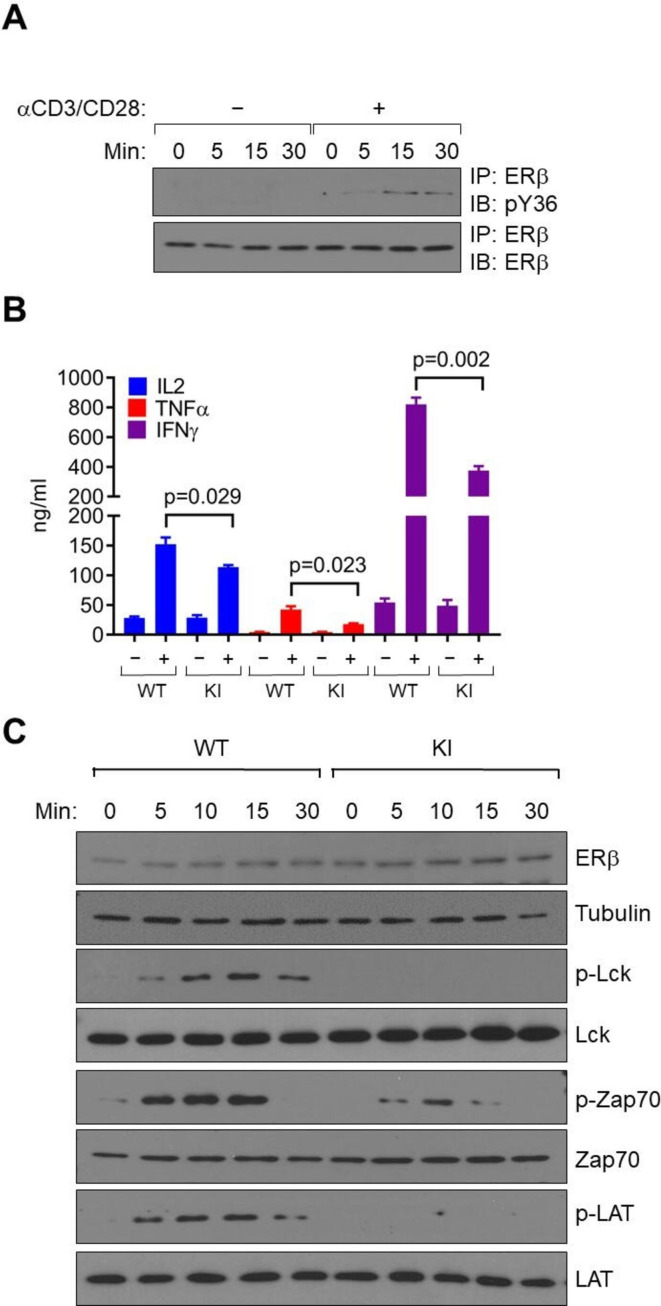
ERβ signaling promotes CD8^+^ T cell activation. (A) IP-Western blot of pY36 (top) and total ERβ (bottom) from mouse primary CD8^+^ T cells with/without anti-CD3/CD28 treatment for the indicated time period. (B) ELISA measurement of cytokine production by purified CD8^+^ T cells from male WT or KI mice, with or without stimulation by anti-CD3/CD28. (C) CD8^+^ T cells from male WT or KI mice were stimulated with anti-CD3/CD28 antibodies for indicated time period and probed for phospho- (p-) and total proteins by immunoblotting. α-tubulin was used as a loading control. IFNγ, interferon-γ; IL2, interleukin 2; KI, knock-in; TNFα, tumor necrosis factor alpha; WT, wild-type.

To elucidate the underlying mechanism by which the ERβ signaling promotes CD8^+^ effector T cell function, we purified primary CD8^+^ T cells from mouse splenocytes and activated them in vitro with anti-CD3/CD28. Y55 phosphorylation of WT ERβ was substantially elevated following T cell activation (figure 3A). Importantly, on anti-CD3/CD28 activation, KI CD8^+^ T cells with abolished ERβ phosphotyrosine switch produced significantly lower antitumor cytokines including IL-2, IFNγ and TNFα than their WT counterparts (figure 3B), further corroborating a cell-autonomous role of ERβ signaling in CD8^+^ T cell function.

Initiation and propagation of TCR signaling involves phosphorylation of several proximal signaling molecules including Lck, Zap70 and LAT, as well as activation of additional downstream signaling molecules.^18^ These post-translational events, which are independent of gene activation at the transcriptional level, occur within minutes following TCR binding to its antigenic ligands. Following anti-CD3/CD28 treatment in vitro, TCR-stimulated phosphorylation of these signaling molecules was readily detectable as early as 5 min following TCR activation (figure 3C). In stark contrast, disabling of the ERβ phosphotyrosine switch markedly attenuated the TCR-triggered signaling cascade in KI CD8^+^ T cells (figure 3C). Given the short time frame of the initial steps in TCR activation (ie, minutes instead of hours), we reasoned that ERβ unlikely facilitates this rapid process through transcriptional (genomic) activation after anti-CD3/CD28 treatment. In support, RNA-seq of primary WT and KI CD8^+^ T cells before in vitro activation did not reveal any significant ERβ-dependent, baseline transcriptomic changes (data not shown). On the other hand, ERβ was detected in both nuclear and membrane-associated subcellular fractions of mouse EL4 lymphoma cells (online supplemental figure S7A). Thus, ERβ signaling promotes rapid activation of TCR signaling cascade of CD8^+^ T cells is likely independent of its canonical transcriptional activity.

10.1136/jitc-2020-001932.supp7 Supplementary data

#### Activation of ERβ signaling by ERβ agonist S-equol enhances αPD-1 immunotherapy

**Figure 4:**
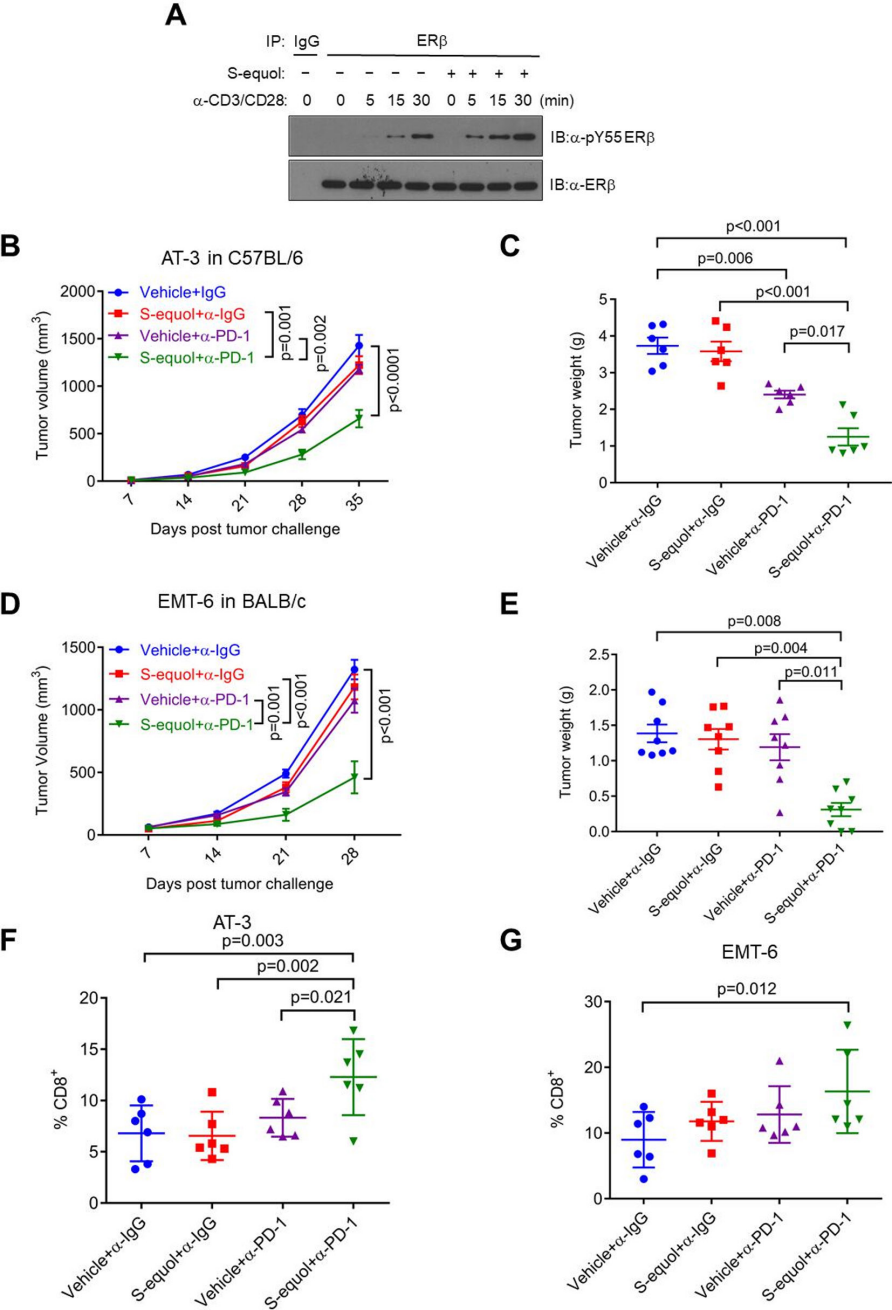
The ERβ agonist S-equol promotes CD8^+^ T cell activation and antitumor immunotherapy. (A) IP-Western blot of pY36 (top) and total ERβ (bottom) in mouse primary CD8^+^ T cells pretreated with S-equol for 4 hours, followed by anti-CD3/CD28 treatment for the indicated time period. (B, C) AT-3 mammary tumor growth (B) and tumor weight (C) in female WT C57BL/6J mice with four-arm treatments. Vehicle+α-IgG, S-equol+α-IgG, Vehicle+α-PD-1, S-equol+α-PD-1. (D, E) EMT-6 mammary tumor growth (D) and tumor weight (E) in female WT BALB/c mice with four-arm treatments. Vehicle+α-IgG, S-equol+α-IgG, Vehicle+α-PD-1, S-equol+α-PD-1. (F, G) Flow cytometry of AT-3 (F) and EMT-6 (G) percentage of CD8^+^ among tumor-infiltrating CD45^+^ cell population. WT, wild-type.

Our published work shows that the ERβ-selective agonist S-equol promotes human ERβ phosphorylation at Y36 in breast cancer cells.^9^ S-equol further enhanced TCR-activated ERβ tyrosine phosphorylation in anti-CD3/CD28-stimulated mouse EL4 lymphoma cells (figure 4A). In light of in vitro data, we assessed a potential effect of S-equol on boosting antitumor efficacy of αPD-1 in mouse tumor models. This experimental design included four arms: (1) IgG isotype control, (2) αPD-1, (3) S-equol and (4) αPD-1+S-equol. In AT-3 and EMT-6 mammary tumor models, tumors were refractory to αPD-1 or S-equol monotherapy, but the combination treatment resulted in appreciable tumor reduction (figure 4B–E). In two additional tumor models (E0771 mammary tumor and B16F10 melanoma), tumors responded to αPD-1 or S-equol monotherapy, and the combination treatment led to even more pronounced tumor reduction than either single-agent treatment (online supplemental figure S7B-E). Consistent with the tumor growth assessment, combination of αPD-1 and S-equol substantially increased tumor-infiltrating CD8^+^ T cells vs control and single agent treatment (figure 4F–G). In aggregate, our data demonstrate the ability of ERβ agonization to sensitize various syngeneic tumors to anti-PD-1 ICB immunotherapy.

To explore clinical correlation between ERβ and antitumor immunity, we used a bioinformatics tool (TIMER) to assess tumor-immune correlations in human tumor samples.^19^ Several solid cancer types display significant positive correlation between ERβ mRNA levels and genes associated with antitumor immunity including CD4, CD8A and GZMB (online supplemental figure S8A-C). Of note, the strongest correlation of ERβ with CD8A and GZMB was observed in breast cancer (online supplemental figure S8B, C). When we compared the immune marker correlation for *ESR1* and *ESR2* specifically in breast cancer, it is striking that unlike *ESR2*, *ESR1* in general exhibits a negative association with these antitumor immune markers (compare online supplemental figure S8D, E), supporting the notion that ERα and ERβ play opposite roles in tumor immune microenvironment. Furthermore, analysis of publicly available breast cancer-related patient survival datasets shows that higher *ESR2* expression levels correlate with longer patient survival, but the correlation is only significant in tumors with relatively abundant tumor-infiltrating CD8^+^ T cells (online supplemental figure S8F, G). Together, these clinical correlations are consistent with our preclinical finding of a prominent role of ERβ signaling in rallying strong antitumor immune response in host CD8^+^ T cells.

10.1136/jitc-2020-001932.supp8 Supplementary data

## Discussion

Pathophysiological significance of ERβ signaling is an important yet historically under-investigated research topic. Despite an increasing appreciation for its anticancer therapeutic potential, there lacks compelling genetic evidence for a definitive role of ERβ signaling in cancer development and progression, which dampers rationalized efforts to mobilize it with targeted precision and synergy. ERβ is implicated in regulation of both tumor and host immune cell functions. For example, ERβ is shown to decrease migration of tumor cells^20^ and increase immune cells trafficking.^21^ Our discovery of an ERβ-specific and tumor-extrinsic ERβ phosphotyrosine switch in antitumor immunity provides a previously unrecognized approach for assessing and rallying ERβ activity in cancer treatment. In particular, the involvement of ERβ signaling in antitumor immunity broadens the horizon for combination therapies that could maximally boost efficacy of ICB immunotherapies and increase survival benefits for more cancer patients. Furthermore, our newly established ERβ KI mouse model provides a powerful tool for further elucidating phospho-ERβ-dependent ERβ functions in normal physiological and disease conditions, and tumor-intrinsic versus extrinsic contexts.

Our previously published work identified c-Abl and EYA2 as the kinase and phosphatase, respectively, that directly and oppositely control ERβ phosphotyrosine status in breast cancer cells.^8^ It remains to be determined whether these molecules directly regulate the ERβ phosphotyrosine switch in CD8^+^ T cells. Of note, c-Abl is implicated in mediating TCR activation.^22^ Furthermore, clinical trials of pharmacological inhibitors of BCR-Abl for solid tumors yielded mixed results, which could be due to the complex action of c-Abl in tumor and host immune cells. Alternatively, ERβ phosphotyrosine in CD8^+^ T cells could be regulated by other tyrosine kinases. We previously screened a mammalian expression library for all known human tyrosine kinases. In addition to c-Abl, Zap70 was also identified as a candidate kinase for human ERβ phosphorylation at the Y36 residue. It is, therefore, conceivable that Zap70 and ERβ could form a positive regulatory loop whereby Zap70 phosphorylates ERβ, which in turn promotes Zap70 association with other signaling molecules in TCR-mediated immune activation.

In addition to full-length ERβ (ERβ1), the human *ESR2* gene also produces several splicing variants (ERβ2–5) with distinct and even opposite biological activities. However, the nature and functions of the corresponding variants for mouse *Esr2* remain unclear. Because all known human variants share the same N-terminal sequence and thus the phosphotyrosine switch, similar putative variants in mouse KI mouse model would be affected as well as the full-length ERβ protein. Future work is warranted to distinguish the effect of the phosphotyrosine switch on full-length and possible splicing variants in human and murine immune cells.

ERβ exerts genomic and non-genomic effects.^7^ We did not observe any transcriptomic changes indicative of genomic actions in ERβ-activated CD8^+^ T cells. Nonetheless, we detected marked defects of mutant T cells in TCR signaling minutes after ERβ activation, supporting non-genomic ERβ actions on T cell activation. In further support of non-genomic ERβ actions on CD8^+^ T cells, we found ERβ in their cell membrane fractions in addition to nuclear localization. Nonetheless, secondary genomic action of ERβ in CD8^+^ T cells is formally possible and not excluded by our studies.

Sex and hormone effects in cancers remain incompletely understood. We previously reported sex differences in tumor growth in a PD-L1-dependent manner^23^ and we found sex differences in other tumors based on obesity and additional factors.^24^ Full understanding of sex differences aside from estrogen effects merit further investigations.

Despite major breakthroughs in anticancer immunotherapies, they are effective only for some patients and usually not curative.^25^ Thus, it is imperative to develop novel stand-alone immunotherapies and/or agents that can improve existing anticancer immunotherapies. Multiple clinical trials have demonstrated human safety of several synthetic and natural ERβ-selective agonists. In particular, S-equol was used in multiple phase I (NCT00787007, NCT00998920, both completed) and phase II clinical trials for the treatment of menopausal symptoms (NCT00962585, completed), benign prostatic hyperplasia (NCT00962390, completed), Alzheimer’s disease (NCT03101085, in progress) and more recently triple negative breast cancer (NCT02352025, early phase I, completed). The antitumor effect of S-equol observed in the current study, either alone or in combination with αPD-1, is consistent with a recent report of another ERβ agonist LY500307 in overcoming tumor resistance to ICB immunotherapy.^6^ Given both tumor-intrinsic and -extrinsic antitumor activities of ERβ, the underlying mechanism by which S-equol inhibits tumor growth could be multifactorial and merits further investigation. Evidently, translating the preclinical findings from our current study into future anticancer clinical trials could lead to novel combination therapies that significantly expand patient populations who can benefit from current immunotherapies.

## Data Availability

All data relevant to the study are included in the article or uploaded as online supplemental information. All relevant data are included in the manuscript.
