# Correction: Estrogen receptor beta signaling in CD8+ T cells boosts T cell receptor activation and antitumor immunity through a phosphotyrosine switch

**DOI:** 10.1136/jitc-2020-001932corr1

**Published:** 2021-10-13

Yuan B, Clark CA, Wu B, *et al*. Estrogen receptor beta signaling in CD8+ T cells boosts T cell receptor activation and antitumor immunity through a phosphotyrosine switch. *J Immunother Cancer* 2021;9:e001932. doi:10.1136/jitc-2020-001932

This article has been corrected since it first published. The provenance and peer review statement has been added.

