# Supplementary figures and images for: Estrogen receptor beta signaling in CD8+ T cells boosts T cell receptor activation and antitumor immunity through a phosphotyrosine switch
Source: J Immunother Cancer. 2021 Jan 18;9(1):e001932. doi: 10.1136/jitc-2020-001932 (PMC7816924; doi:10.1136/jitc-2020-001932)

Supplementary Figure S1. Generation of ER $\beta$  Y55F knockin mice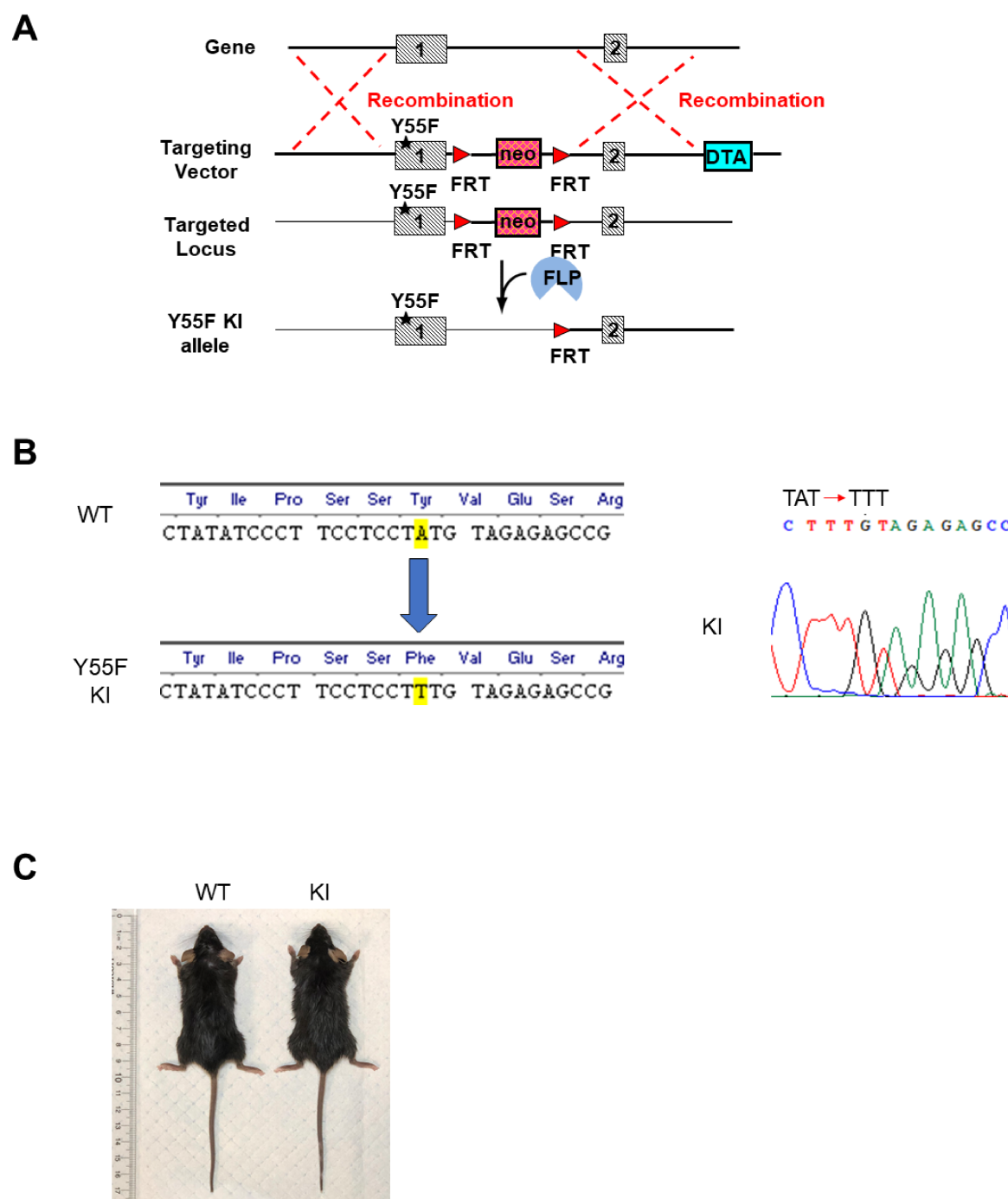

Supplement: Supplementary data [file jitc-2020-001932supp001.pdf]

Supplementary Figure S3. immunophenotyping analysis of tumor from WT and KI mice

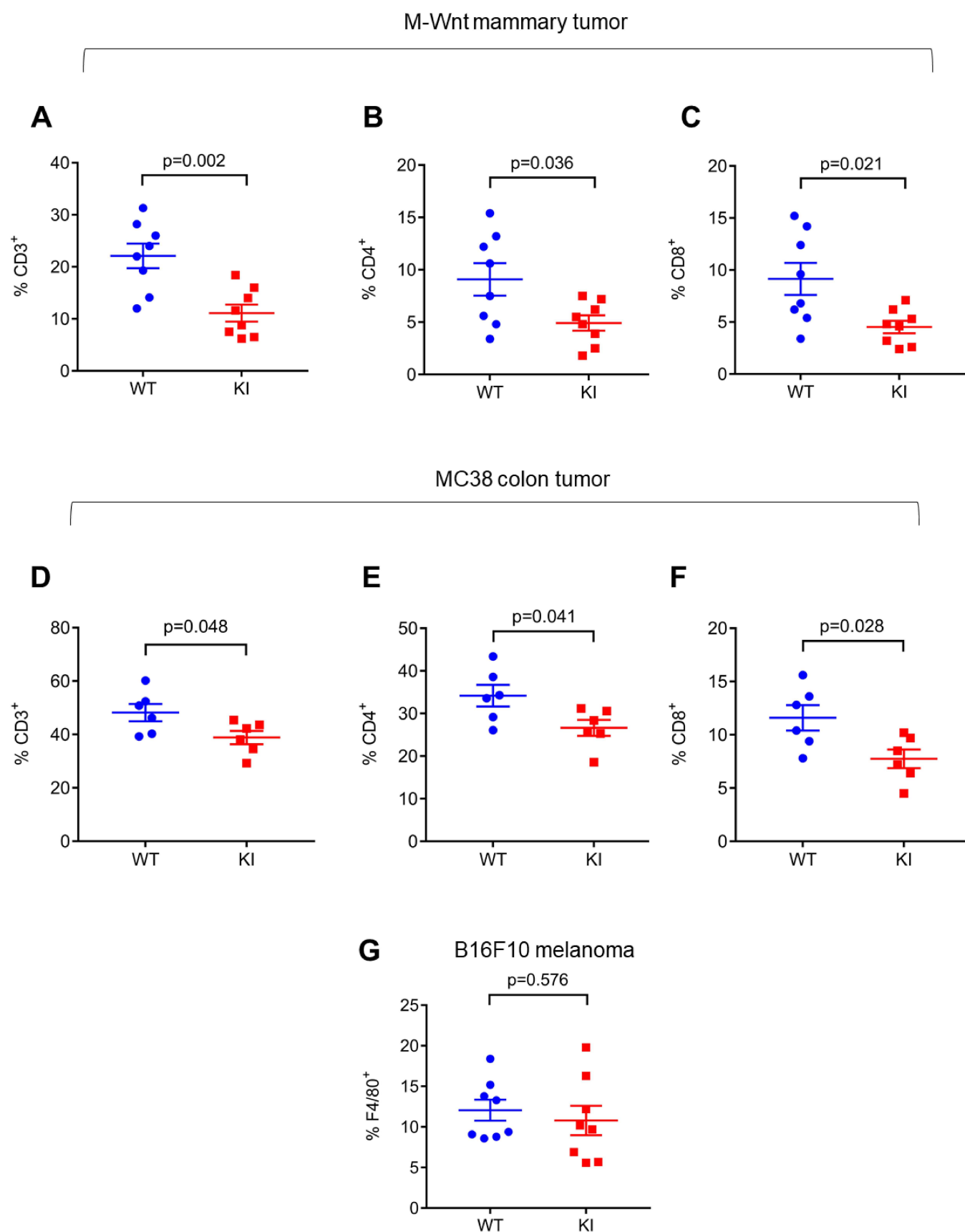

Supplement: Supplementary data [file jitc-2020-001932supp003.pdf]

Supplementary Figure S5. Tumor growth in chimeric mice reconstituted with WT or KI bone marrow

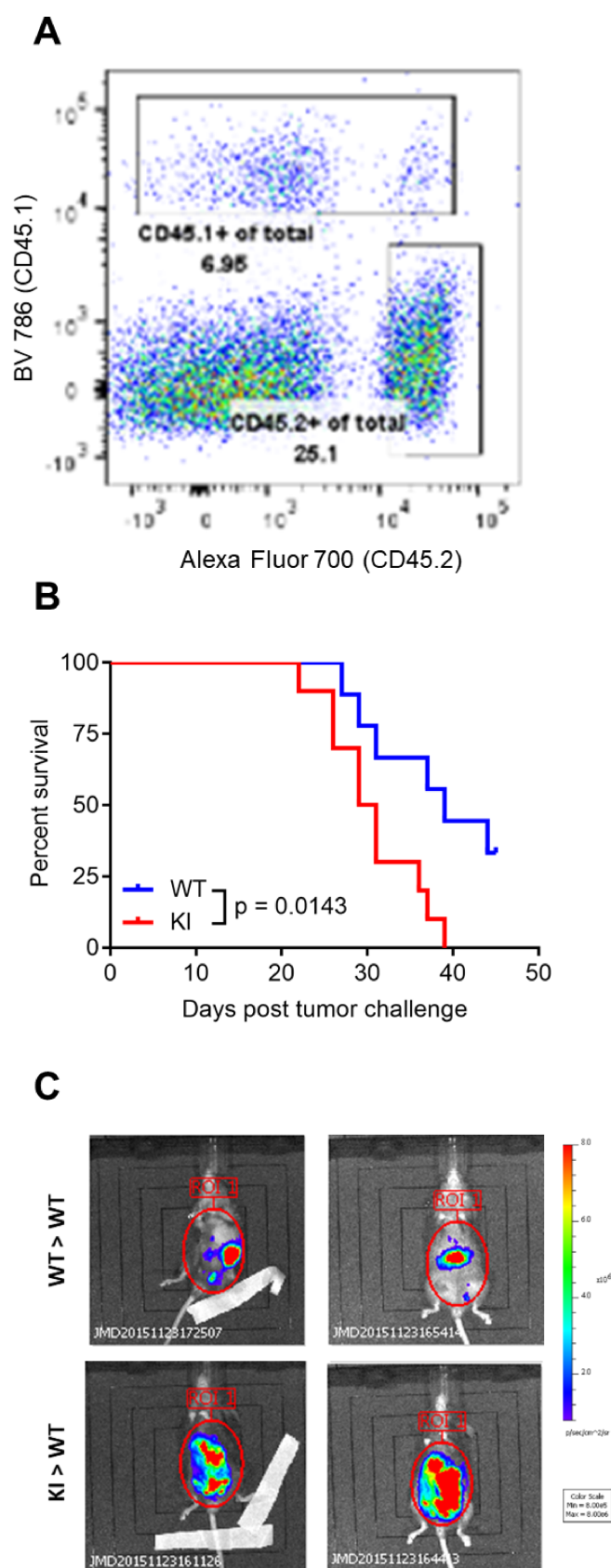

Supplement: Supplementary data [file jitc-2020-001932supp005.pdf]

Supplementary Figure S6. Immunophenotyping of tumors from chimera mice

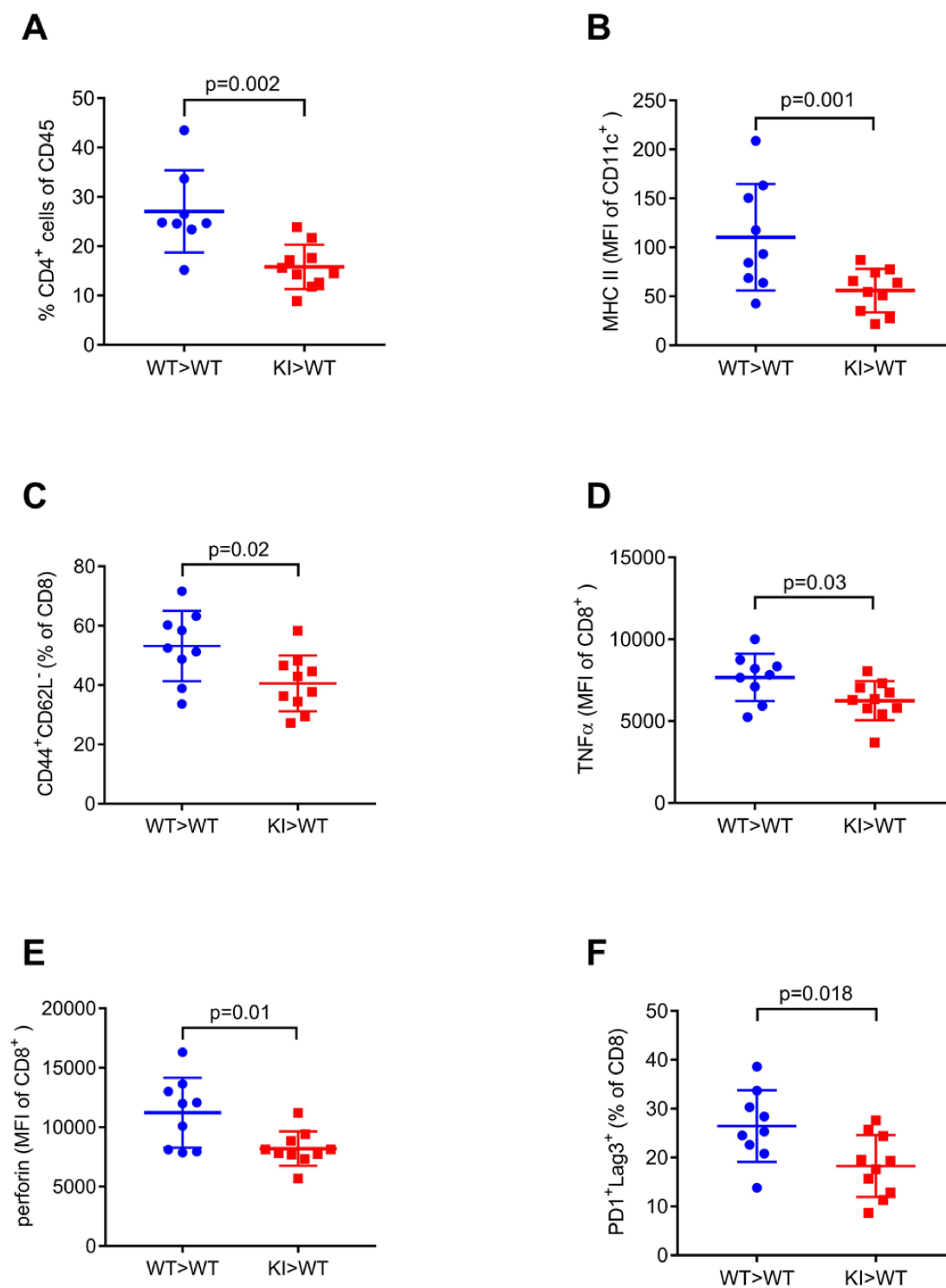

Supplement: Supplementary data [file jitc-2020-001932supp006.pdf]
