# Supplementary material for: Estrogen receptor beta signaling in CD8+ T cells boosts T cell receptor activation and antitumor immunity through a phosphotyrosine switch
Source: J Immunother Cancer. 2021 Jan 18;9(1):e001932. doi: 10.1136/jitc-2020-001932 (PMC7816924; doi:10.1136/jitc-2020-001932)

Supplementary Figure S2. The phosphotyrosine switch is important for host ERβ to inhibit tumor growth

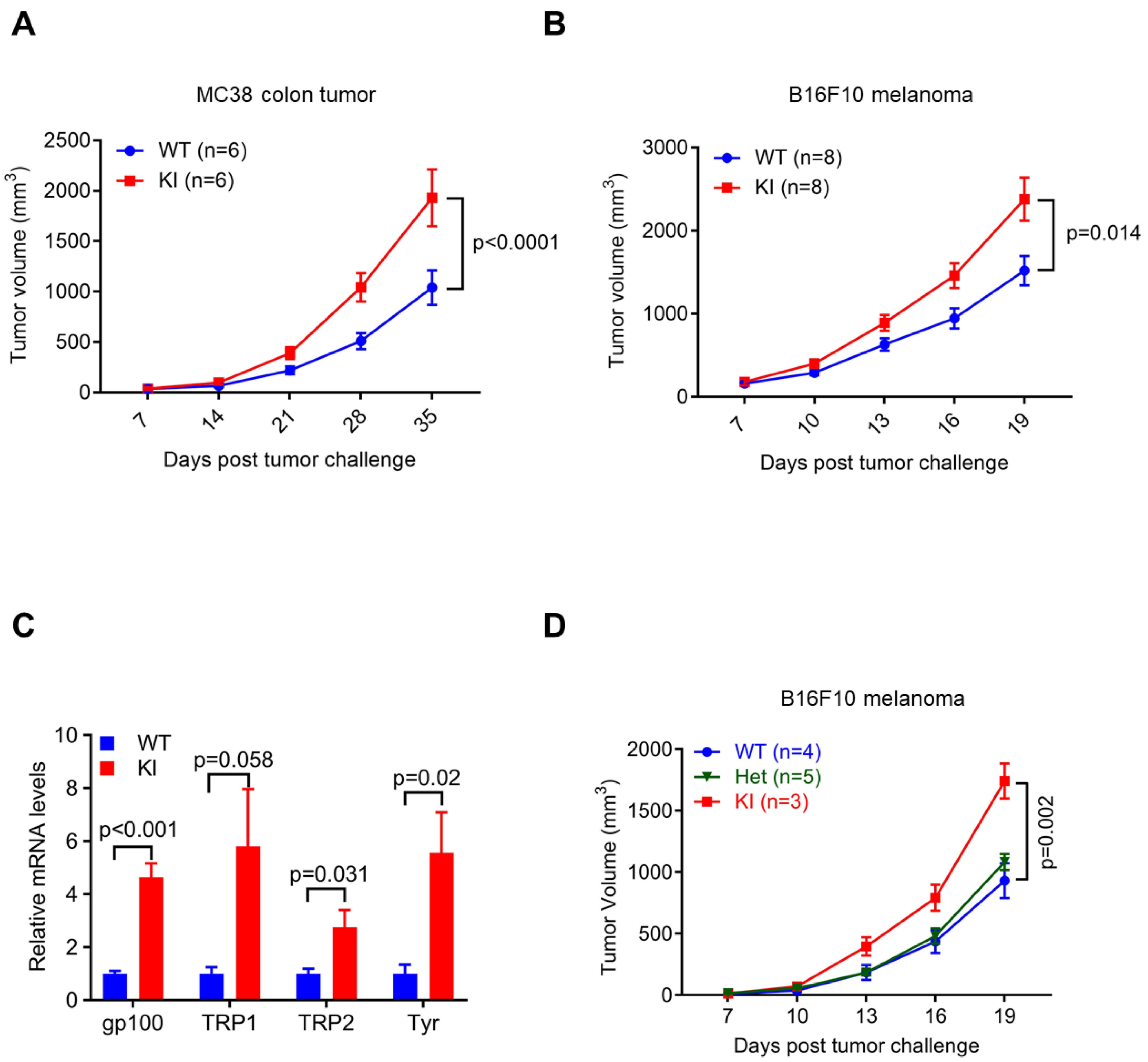

Supplement: Supplementary data [file jitc-2020-001932supp002.pdf]
