# Supplementary material for: Estrogen receptor beta signaling in CD8+ T cells boosts T cell receptor activation and antitumor immunity through a phosphotyrosine switch
Source: J Immunother Cancer. 2021 Jan 18;9(1):e001932. doi: 10.1136/jitc-2020-001932 (PMC7816924; doi:10.1136/jitc-2020-001932)

Supplementary Figure S7. ERβ-selective agonist S-equol boosts anti-PD-1 immunotherapy

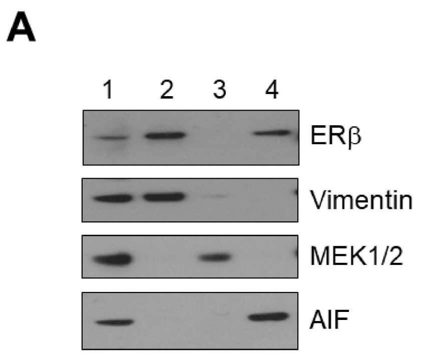

1. whole cell lysate;
2. nuclear and cytoskeleton;
3. cytoplasmic;
4. membrane associated.

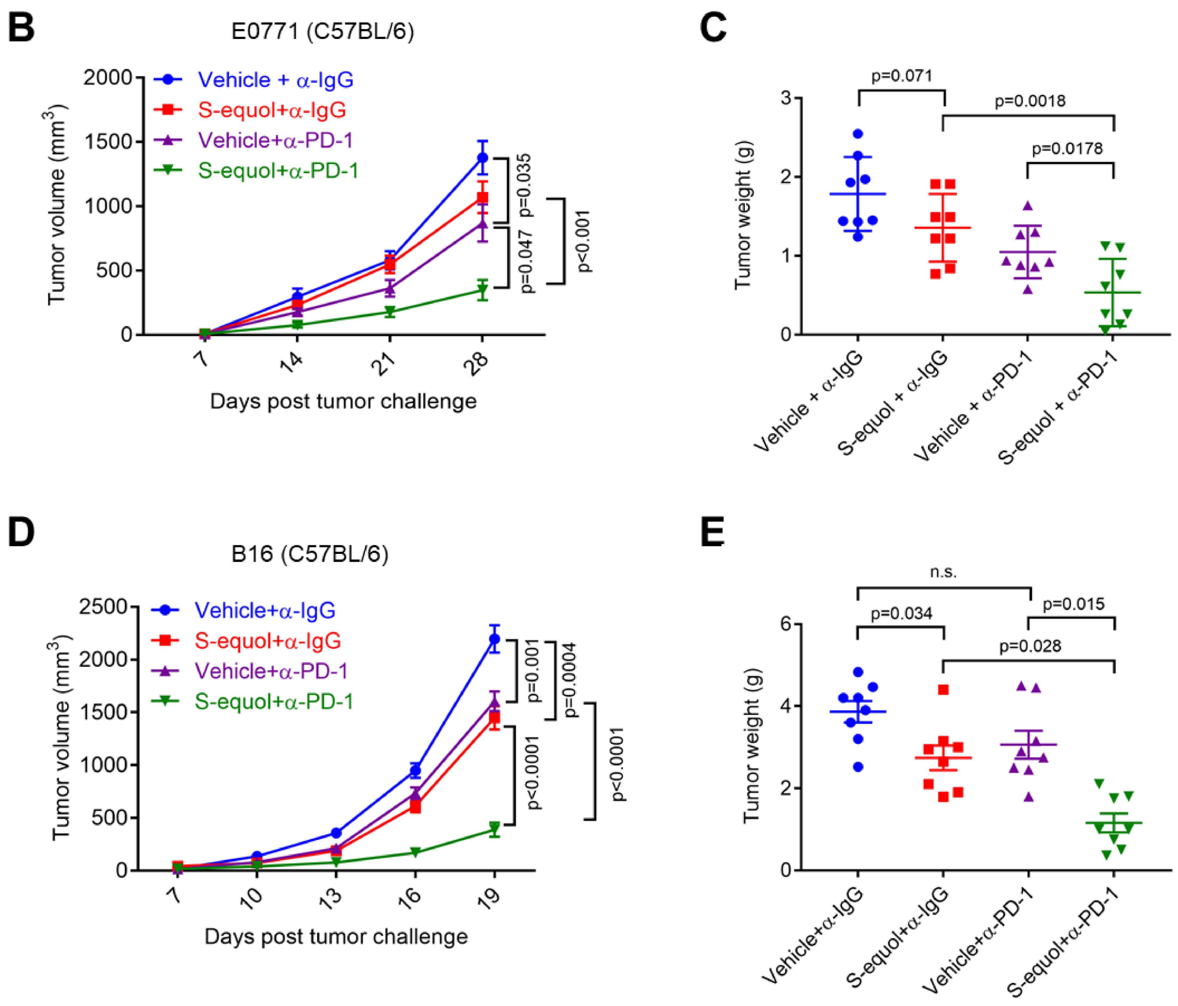

Supplement: Supplementary data [file jitc-2020-001932supp008.pdf]
